# Effect of Counterions on the Interaction among Concentrated Spherical Polyelectrolyte Brushes

**DOI:** 10.3390/polym13121911

**Published:** 2021-06-08

**Authors:** Yunwei Wang, Li Li, Yiming Wang, Qingsong Yang, Zhishuang Ye, Liang Sun, Fan Yang, Xuhong Guo

**Affiliations:** 1 State Key Laboratory of Chemical Engineering, Engineering Research Center of Large Scale Reactor Engineering and Technology (Ministry of Education), and International Joint Research Center of Green Energy Chemical Engineering, East China University of Science and Technology, Shanghai 200237, China; yunweiwang@mail.ecust.edu.cn (Y.W.); yimingwang@ecust.edu.cn (Y.W.); yangqs2013@163.com (Q.Y.); yzsecust@163.com (Z.Y.); yangf@ecust.edu.cn (F.Y.); 2 Engineering Research Center of Xinjiang Bingtuan of Materials Chemical Engineering, Shihezi University, Shihezi 832000, China; sunliang055x@163.com

**Keywords:** spherical polyelectrolyte brush, counterions, WAXS, rheology, SAXS

## Abstract

The effect of counterions on interactions among spherical polyelectrolyte brushes (SPBs) was systematically investigated by rheology, small-angle X-ray scattering (SAXS) and wide-angle X-ray scattering (WAXS). The SPB particles consist of a solid polystyrene (PS) core with a diameter of ca.100 nm and a chemically grafted poly-(acrylic acid) (PAA) brush layer. Metal ions of different valences (Na^+^, Mg^2+^ and Al^3+^) were used as counterions to study the interactions among concentrated SPBs. The so-called “structure factor peak” in SAXS, the “local ordered structure peak” in WAXS and rheological properties indicated the interactions among concentrated SPBs. Combining SAXS, WAXS and rheology, the formation mechanism of the local ordered structure among PAA chains in the overlapped area of adjacent SPB, which was generated due to the bridge function of counterions, was confirmed. In contrast, excessive counterions shielded the electrostatic interaction among PAA chains and destroyed the local ordered structure. This work enriches our understanding of the polyelectrolyte assembly in concentrated SPBs under the effect of counterions and lays the foundations for SPB applications.

## 1. Introduction

Spherical polyelectrolyte brushes (SPB) are nanoparticles that consist of a spherical hydrophobic polymer core and are grafted with a linear hydrophilic polyelectrolyte shell on the surfaces of spherical core substrates [1,2,3,4]. In the field of material science and colloid science, SPBs show great potential an broad prospects [5,6,7,8,9]. Similar with other polyelectrolyte aqueous dispersions, the interactions among concentrated SPBs are highly determined by the properties of SPB dispersions [10]. Interestingly, the properties of SPBs can be simply designed and adjusted by choosing different synthesis conditions and different monomers [11,12]. Additionally, the lengths of poly(acrylic acid) (PAA) brush chains are strongly affected by environmental conditions. Therefore, the interactions among SPBs composed of weak polyelectrolyte can be affected by pH values and the ionic strength in the system [13,14].

Recently, SPBs dispersed in dilute solutions have been thoroughly investigated, with studies focusing on their properties and applications [15,16,17]. For PAA brushes, the polyelectrolyte chains of SPB swell in aqueous dispersions with an increase in pH according to the Daoud–Cotton theory and the mean field theory [18,19], which proposes that there are interactions among concentrated SPBs and the charge degree of PAA chains. A number of characterization methods have been used to characterize the properties of SPBs, such as WAXS, [20] SAXS [21,22,23,24,25], scanning electron microscopy (SEM) [26,27], transmission electron micrography (TEM) [28,29], dynamic light scattering (DLS) [30] and small-angle neutron scattering (SANS) [31]. However, the interactions among concentrated SPB aqueous dispersions have not been carefully examined or investigated by researchers, and there is a scarcity of reports on this in the literature. The viscosity of SPB dispersions, especially at high concentrations, is strongly affected by counterions. Therefore, to investigate the effect of counterions on the interaction among concentrated spherical polyelectrolyte brushes is of critical importance for their applications in coating and painting. 

**Figure 1:**
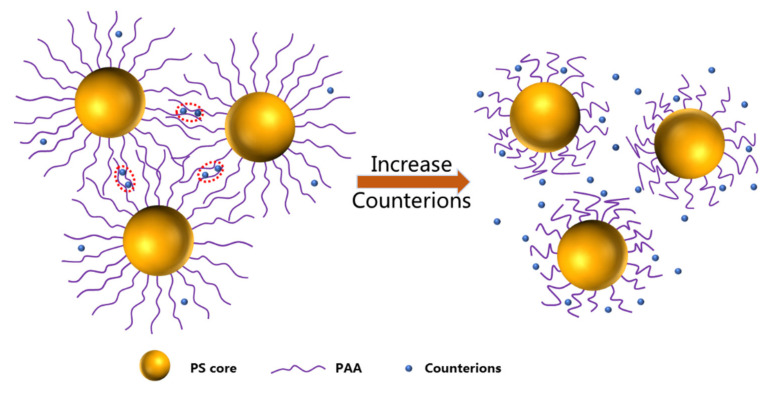
Schematic illustration of interaction among concentrated SPBs with increase in counterion concentration.

Very recently, we studied PAA brushes in different SPB concentrations, pH values and salt concentrations [32]. Firstly, with the increase in SPB concentration, the viscosity of SPB aqueous solution significantly rose. Then, a “polyelectrolyte peak” [32] at the high *q* range was observed in the SAXS curve. This was assumed to be caused by the formation of local ordered structures among overlapped polyelectrolyte chains. Moreover, the strength of the polyelectrolyte peak was clearly affected by pH and counterions. To provide more details of this phenomenon, the effects of counterions on the interactions among concentrated SPBs were systematically investigated by combining SAXS, WAXS and rheology in this paper. Metal ions with different valences (Na^+^, Mg^2+^ and Al^3+^) were used in this experiment, and an interesting regularity was discovered. As show in Figure 1, the addition of counterions leads to local ordered structures among polyelectrolyte chains. In this case, the counterions play the role of a bridge to connect the polyelectrolyte chains. However, further increase in the concentration of counterions results in the disappearance of such local ordered structures, mainly because the counterions shield the electrostatic interaction among polyelectrolyte chains. To analyze this local ordered structure, concentrated SPB aqueous dispersions were characterized by SAXS, which can reveal the strength of interaction by structure factor in the SAXS curve. WAXS has also been proved to be an effective way to analyze local ordered structures [33,34,35]. SAXS and WAXS are microscopic characterization methods. At the macroscopic level, the rheological properties of SPB aqueous dispersions with different metal ions were studied to further certify the local ordered structures among SPBs. Combining rheology, WAXS and SAXS, the effect of different valence metal ions on the interactions among concentrated SPBs was systematically studied. This research enriches the polyelectrolyte theory and greatly accelerates the applications of SPBs.

## 2. Experimental Section

### 2.1. Materials

Potassium persulfate (KPS) was purchased from Shanghai reagent company (Shanghai, China) Co., Ltd. and was purified by recrystallization. Styrene and acrylic acid were also bought from SRC, and their inhibitors were removed by reduced pressure distillation. Sodium chloride (NaCl), magnesium chloride (MgCl_2_) and aluminum chloride (AlCl_3_) were purchased from Sinopharm Chemical Reagent (Shanghai, China) Co., Ltd. The photo-initiator 2-[p-(2-hydroxy-2-methyl-propiophenone)]-ethylene glycol methacrylate (HMEM) was synthesized, and the synthesis process is described in a previous work^6^. Sodium hydroxide (NaOH; SRC), sodium dodecyl sulfate (SDS; SRC) and other chemicals were used as received without further purification. The used water was obtained and purified by reverse osmosis of Millipore Milli-Q.

### 2.2. Preparation of SPBs

The synthesis of SPBs was performed according to our previous work by photo-emulsion polymerization.^1^ First, the PS core was synthesized by conventional emulsion polymerization. KPS (0.37 g) as initiator and SDS (0.24 g) as surfactant were dissolved in 75 mL deionized water. Then, 5 g of styrene was added into the above mixture under nitrogen atmosphere with a constant stirring rate of 300 rpm. The reaction was carried out at 80 °C for two hours. Second, the reaction temperature was adjusted to 70 °C, and the HMEM (0.5 g), which was dissolved in 3.5 g acetone, was slowly added to the three-necked flask under the “starved” condition (1 drop per 6–7 s). After another 2.5 h, a photoinitiator shell with a thickness of 2–3 nm was formed on the PS surface by copolymerization. The PS aqueous solution was cooled to 25 °C, and the impurities were removed by ultrafiltration with ultrapure water. Finally, the diluted PS core (50 g) dispersion mixed with the AA monomer (0.75 g, 100 mol% of styrene) was added to a photoreactor. Using photoemulsion polymerization to synthesize SPB dispersion, the experiment was carried out under UV irradiation with gentle magnetic stirring. After 2.5 h, the PAA chains were densely grafted from the surface of PS cores and formed the SPB dispersions. The obtained SPB dispersions were purified by ultrafiltration with ultrapure water approximately ten times. The pH of SPB dispersion was adjusted by NaOH and HCl, and the concentration of counterions was adjusted by NaCl, MgCl_2_ and AlCl_3_.

### 2.3. Characterization

SAXS and WAXS characterization was carried out using a BL16B1 beamline at the Shanghai Synchrotron Radiation Facility (SSRF). The distance of sample to the detector was set as 0.2 m in the WAXS experiment and 5 m in the SAXS experiment. The incident wavelength of X-ray was 1.24 nm, and the scattering information of the different sample was recorded by the 2D detector (MAR165 CCD). Then, using the software (Fit 2 D) to obtain the one-dimensional curve of SAXS and WAXS, the scattering vector q range was set from 0.05 to 25 nm^−1^. In these q ranges, the sufficient information of the SPB dispersion could be acquired. All of the data deduct the background of the water and the empty sample. The rheological properties of the SPBs were measured with a stress-controlled rotational rheometer (Physica MCR 501, Anton Paar). The temperature was set with an accuracy of 0.05 °C. The viscosity and shear rate of the concentrated SPBs were measured after a pre-shearing of 100 s^−1^ for 1 min and with a waiting time of 10 s. The frequency dependence of the storage modulus G′ and loss modulus G″ were measured after a 1 min pre-shear at a shear rate of 100 s^−1^ and with a 10 s waiting time for the 1% strain from 10 to10^−3^ Hz. The pH was measured with a DELTA 320 pH-meter. For all of the samples in the experiment, the concentration of SPBs was 12 wt%, and the pH was adjusted to 7.0.

### 2.4. SAXS Theory

The data analysis in this work was conducted following recently reported studies. [23,24]. The scattering vector *q* was correlated with the scattering angle *θ* according to the following equation:

(1) $$q=\left(4\pi /\lambda \right)sin\left(\theta /2\right)$$

where *θ* is the scattering angle and λ is the wavelength of incident X-ray radiation (1.24 nm). The scattering intensity *I*(*q*) can be described as follows:

(2) $$I\left(q\right)=NI_{0}\left(q\right)S\left(q\right)$$

where *N* is the number density, *I*_0_(*q*) is the scattering intensity of an isolated particle and *S*(*q*) is the structure factor that indicates the strength of the interaction among particles. 

Previous articles [26,27,28] have proved that the structure factor *S*(*q*) is only obvious at the smaller *q* region for systems of concentrated spherical particles. For the dilute solution (<1 wt%), the structure factor can be set as constant, i.e., *S*(*q*) = 1, suggesting that the scattering curves directly represent the form factors of the particles. The interaction among particles can be ignored in the analysis. In this way, the structure factor *S*(*q*) can be expressed by the following equation:

(3) $$S\left(q\right)=I\left(q\right)/NI_{0}\left(q\right)$$

For the SPB dispersion, the scattering intensity of an isolated SPB can be decomposed into three independent parts as the following equations:

(4) $$I_{0}\left(q\right)=I_{cs}\left(q\right)+I_{PS}\left(q\right)+I_{fluct}\left(q\right)$$

where *I_cs_*(*q*) signifies the contribution of the overall core–shell structure of the particles, which is caused by the contrast of the electron density between the core–shell structure and the surrounding medium. For the SPB dispersion, SPBs are spherical particles, *I_cs_*(*q*) is equal to the square of the scattering amplitude B(q), and the scattering amplitude *B*(*q*) can be described as shown below:

(5) $$B\left(q\right)=\int b(\rho \left(r\right)-\rho \left(m\right)r^{2}\frac{\sin qr}{qr}dr$$

where *b* is the Thomson scattering length (0.282 × 10^−14^ m), ($\rho \left(r\right)-\rho \left(m\right)$) denoting the radial excess electron density along the radial distance r. For SPB dispersion, ($\rho \left(r\right)-\rho \left(m\right)$) is the contrast between the radial electron density of the SPB $\rho \left(r\right)$ and the electron density of ultrapure water $\rho \left(m\right)$ since water is used as solvent. 

**Figure 2:**
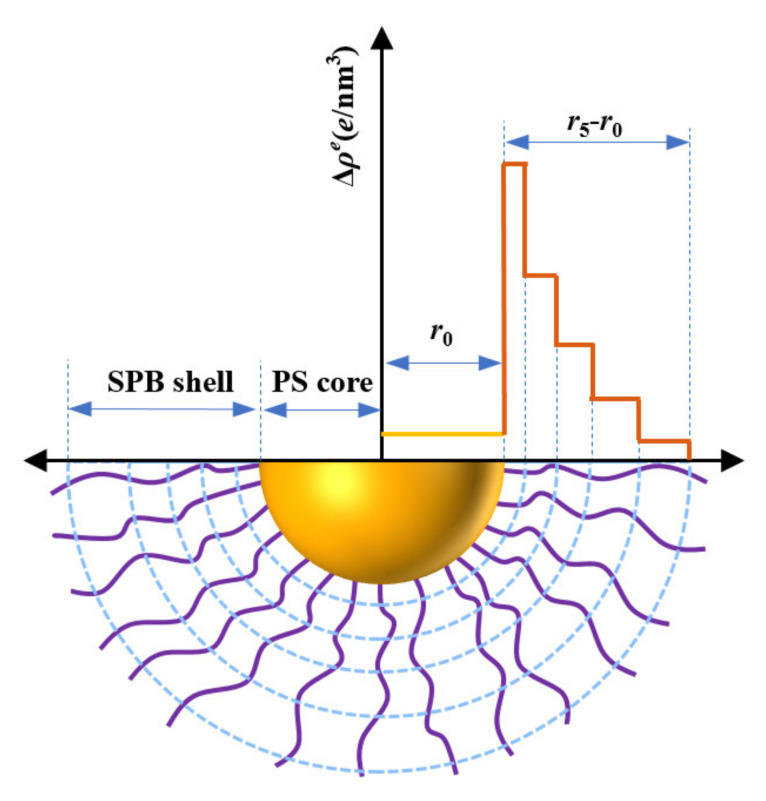
Five-layer fitting model of SPBs.

According to previous work of our group, the “five-layer” model was chosen to fit the SAXS data, which has already been proven to be a feasible theoretical distribution model for the SPB system [36]. As shown in Figure 2 [37,38], the radius of the PS core is set as $r_{0}$, and the PAA shell is radially divided into five layers, with $r_{i}$ (1≤i≤5) representing the outer radius of the PAA layer. Thus, on the basis of the five-layer model, $B\left(q\right)$ of SPBs can be calculated by summing up the scattering amplitudes of the PS core and each layer of the PAA shell. 

The second term $I_{PS}\left(q\right)$ of Equation (4) is the scattering intensity resulting from the density fluctuations of the PS core, which is much smaller than the other two parts. Thus $I_{PS}\left(q\right)$ could be ignored in our research.

The final term $I_{fluct}\left(q\right)$ of Equation (4) results from the thermal fluctuations of the PAA chains, which can be determined by the following equation:

(6) $$I_{fluct}\left(q\right)=\frac{I_{fluct}\left(0\right)}{1+\xi^{2}q^{2}}$$

In this equation, $I_{fluct}\left(0\right)$ is treated as adjustable parameters in our analysis, and ξ is the correlation length of the spatial fluctuations of the PAA chains, which is of the order of a few nanometers. For SPB dispersion, $I_{fluct}\left(q\right)$ only influence scattering intensity *I*(*q*) at high scattering angles (q>0.5 nm^−1^). 

## 3. Results and Discussion

### 3.1. SAXS 

SAXS measurements were conducted on SPB dispersions with salt concentrations of 0.01, 0.1, 1, 5, 10, 50 and 100 mM. The SPB concentration and the pH were kept constant at 12 wt% and 7, respectively, and the five-layer model (Figure 2) was used to fit and analyze the SAXS data in this work. The structure factor S(*q*) was obtained according to Equation (3).

**Figure 3:**
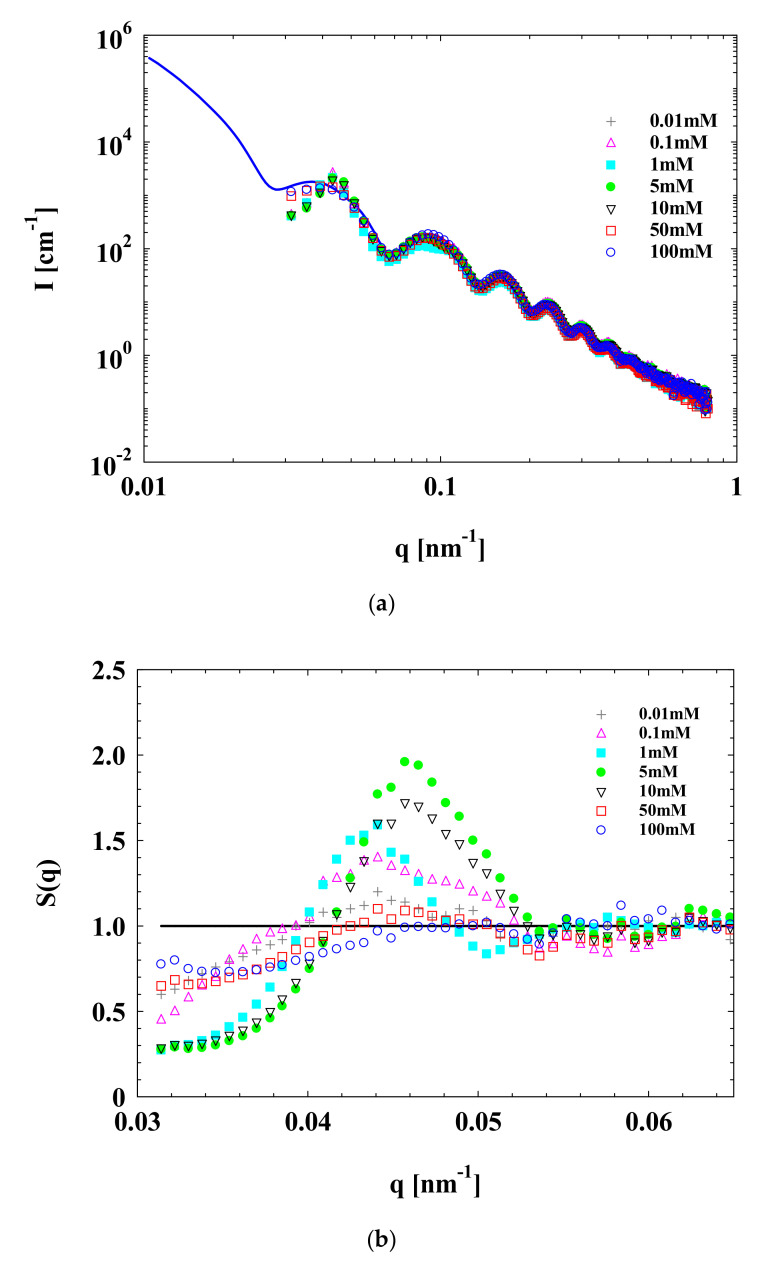
SAXS data of concentrated SPB dispersion at various Na^+^ concentrations. SPB concentration = 12 wt%, pH = 7. (**a**) Scattering intensity *I*(*q*) of SPBs as a function of scattering angle *q*; (**b**) structure factor S(*q*) of concentrated SPB dispersion as a function of scattering angle *q*.

SAXS scattering curves of SPB with different concentrations of Na^+^ are shown in Figure 3. It is obvious that when the Na^+^ concentration increased from 0.01 to 5 mM, the maximum of the structure factor *S*(*q*) (e.g., at *q* around 0.046 nm^−1^) continued to grow monotonically but decreased when Na^+^ concentration further increased from 5 to 100 mM. We named this peak in the *S*(*q*) curve the “structure factor peak”. The increase in the maximum of SAXS data means that there is an increase in interactions among concentrated SPB chains. This can be attributed to the enhancement of counterions, which leads to more ionized carboxyl groups in PAA chains via the increase in Na^+^ concentration from 0.01 to 5 mM. Although the pH value of aqueous SPB dispersion was adjusted to 7, there were still non-dissociative carboxyl groups, and the PAA chains were not fully stretched according to the literature [2,38,39]. In this situation, the counterions played the role of a bridge to connect the concentrated PAA chains forming a local ordered structure. However, a further increase in Na^+^ concentration would lead to the *S*(*q*) approaching a constant (*S*(*q*) = 1), indicating that the interactions among concentrated SPBs become smaller. This could be because excess counterions shield the electrostatic interactions among the PAA chains, resulting in their shrinkage. 

**Figure 4:**
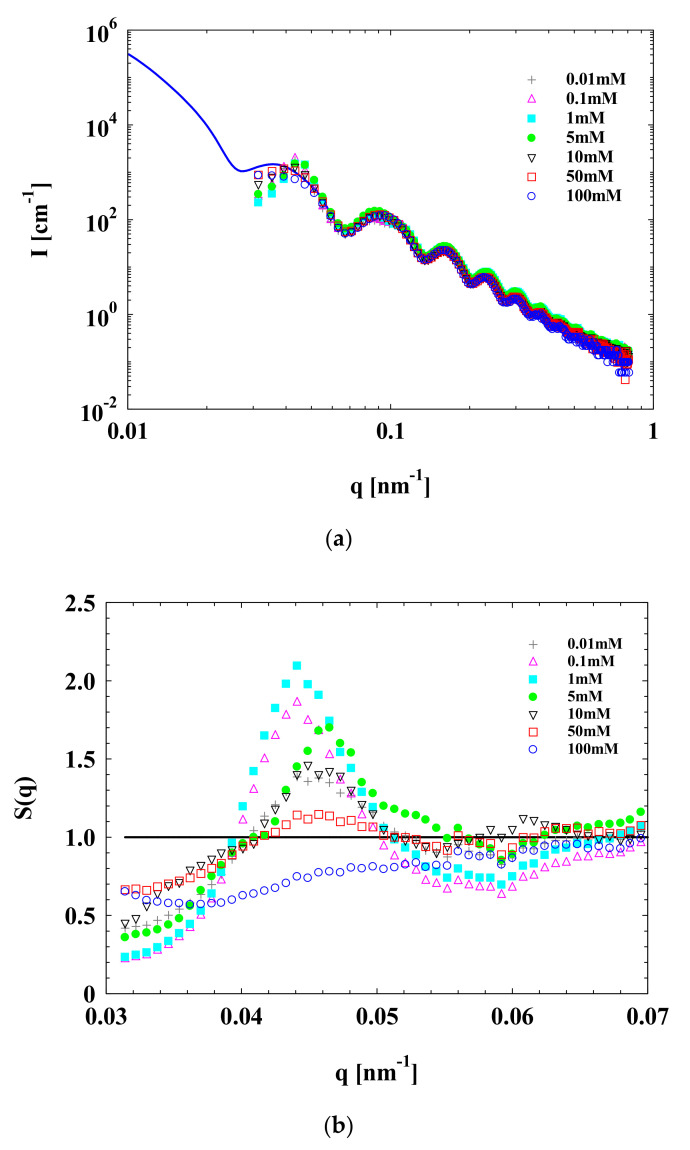
SAXS data of concentrated SPB dispersion at various Mg^2+^ concentrations. SPB concentration = 12 wt%, pH = 7. (**a**) Scattering intensity *I*(*q*) of SPB as a function of scattering angle *q*; (**b**) structure factor S(*q*) of concentrated SPB dispersion as a function of *q*.

To explore the effects of Mg^2+^ concentration on the interactions among concentrated SPBs, SPB dispersions with a concentration of 12 wt% and various Mg^2+^ concentrations (0.01, 0.1, 1, 5, 10, 50 and 100 mM) were characterized by SAXS (Figure 4). The pH value was maintained at 7. 

Different to the effect of Na^+^, with the elevation of Mg^2+^ concentrations from 0.01 to 1 mM, the structure factor *S*(*q*) in the SAXS curve underwent an initial increase and then reached a maximum at the Mg^2+^ concentration of 1 mM. For the Na^+^ ions, however, the maximum *S*(*q*) was achieved at the concentration of 5 mM. This means that the influence of divalent salt ions (Mg^2+^) on the interaction among concentrated SPBs is more significant than that of monovalent salt ions (Na^+^). The reason for the formation of the structure factor peak is similar to that of the polyelectrolyte peak [32] according to our previous work. Both of these peaks indicate the strength of the local ordered structure among the PAA chains. 

This non-monotonic variation of structure factor with a counterion concentration can be explained by a partial replacement of protons within the SPBs with increased counterions^7^. When the Mg^2+^ concentration changed from 0.01 to 1 mM, the dissociation of carboxyl groups increased and led to the stretching of PAA chains, because Mg^2+^ redistribution plays an important role. Moreover, with the further increase in Mg^2+^ concentration to 100 mM, the structure factor peak decreased, in line with the change in the polyelectrolyte peak. This is believed to be caused by excessive Mg^2+^, which screens the electrostatic interactions among SPBs [7,10] and breaks the local ordered structure among PAA chains.

**Figure 5:**
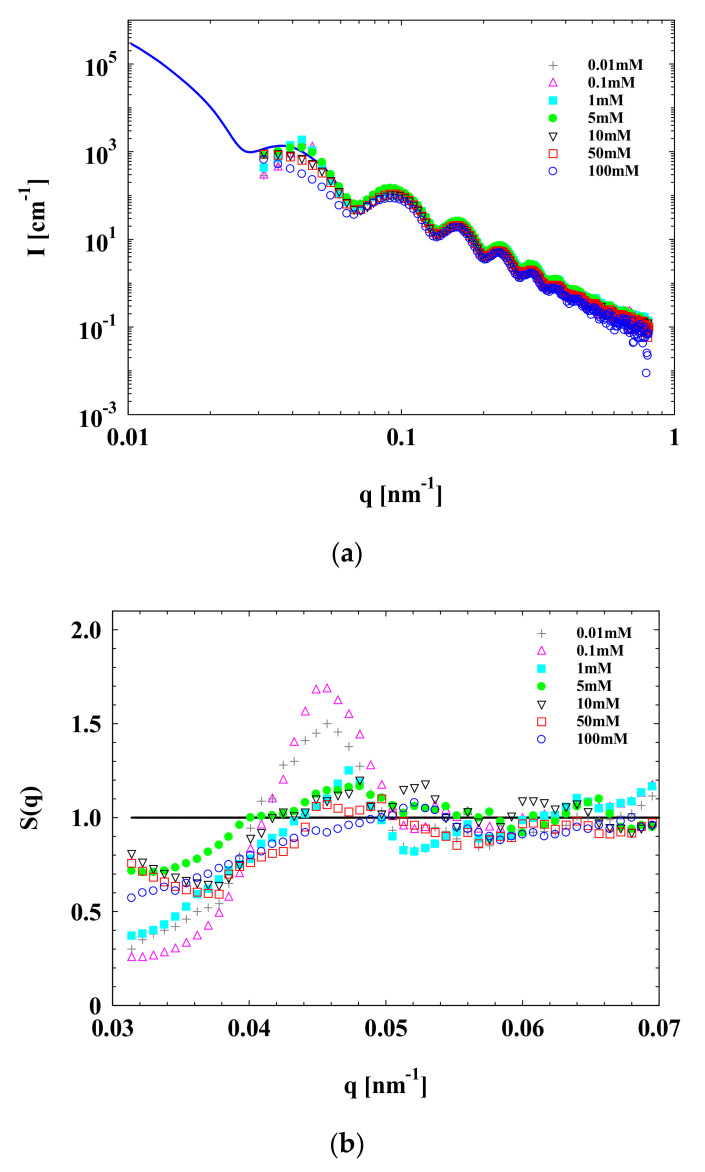
SAXS data of concentrated SPB dispersion at various Al^3+^ concentrations. SPB concentration = 12 wt%, pH = 7. (**a**) Scattering intensity *I*(*q*) of SPBs as a function of scattering angle *q*; (**b**) structure factor *S*(*q*) of concentrated SPB dispersion as a function of *q*.

Figure 5 shows the relationship between the SAXS data of the SPB solution and the *q* value at various Al^3+^ concentrations. Unlike Na^+^, both Mg^2+^ and Al^3+^ have high valence. Thus, they are more likely to cause a bridge among PAA chains and can easily shield electrostatic interactions. As shown in Figure 5, when the Al^3+^ concentration reached 0.1 mM, the strength of the structure factor peak reached its maximum and then began to decrease. On the one hand, the Al^3+^ had a stronger electrostatic shielding effect, while the ion radius of Al^3+^ was much larger than that of Na^+^ and Mg^2+^; thus, it was more difficult for Al^3+^ to enter the middle of the PAA chains due to steric hindrance. 

### 3.2. WAXS

A large number of studies [40,41,42] have confirmed that WAXS is a powerful and effective means to characterize the local ordered structure of polyelectrolyte chains. Here, WAXS was used to characterize the influences of different valences and concentrations of metal ions on the local ordered structure of PAA chains. 

**Figure 6:**
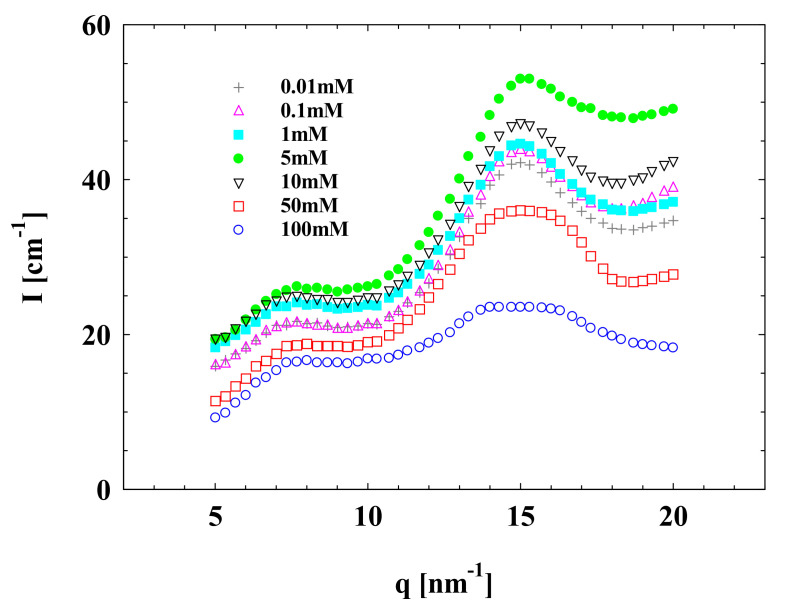
WAXS data of concentrated SPB dispersion at different Na^+^ concentrations. SPB concentration = 12 wt%, pH = 7.

For Na^+^, interestingly, an extra peak appeared at *q* around 16 nm^−1^ in the WAXS spectra (Figure 6) when the SPB concentration increased to 12 wt%. This extra peak was named the “local ordered structure peak”. The appearance of the local ordered structure peak is due to the formation of local ordered structure among PAA chains from neighbored SPBs. When compared to the WAXS curve of different Na^+^ concentrations in Figure 6, the peak at *q* around 16 nm^−1^ should result from the local ordered structure formed by overlapped PAA chains among the neighbored SPBs bridged by counterions under the driven forces of electrostatic interactions, which is similar to the *S*(*q*) curve observed by SAXS. However, with a further increase in the Na^+^ concentration from 5 to 100 mM, the height of the local ordered structure peak in WAXS spectra was significantly reduced. It seems that the arrangement of PAA chains among SPBs is relatively stable and forms a net-like ordered structure. Excessive sodium ions gradually destroy this structure.

**Figure 7:**
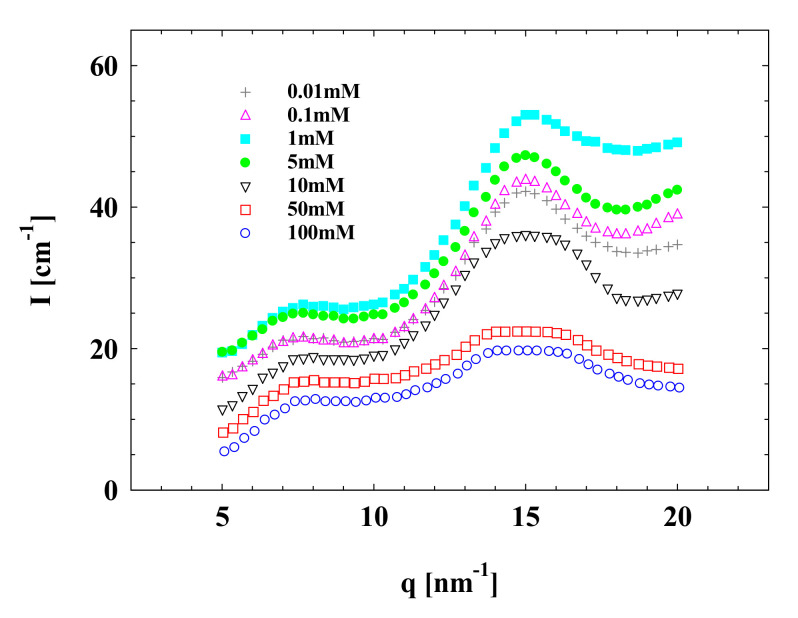
WAXS data of concentrated SPB dispersion at different Mg^2+^ concentrations; SPB concentration = 12 wt%, pH = 7.

Analogous to the SAXS data as shown above, a similar change in the local ordered structure peak at around 16 nm^−1^ in the WAXS spectrum was observed, as shown in Figure 7. However, both occurred at a low Mg^2+^ concentration of 0.01 mM. At a relatively high concentration of 100 mM, the interactions among SPBs seem to be decreased, leading to the unremarkable local ordered structure peak. Only at the moderate salt concentration of 1 mM Mg^2+^ can a significant local ordered structure peak be found in WAXS spectra, suggesting the formation of ordered structure among SPBs. 

**Figure 8:**
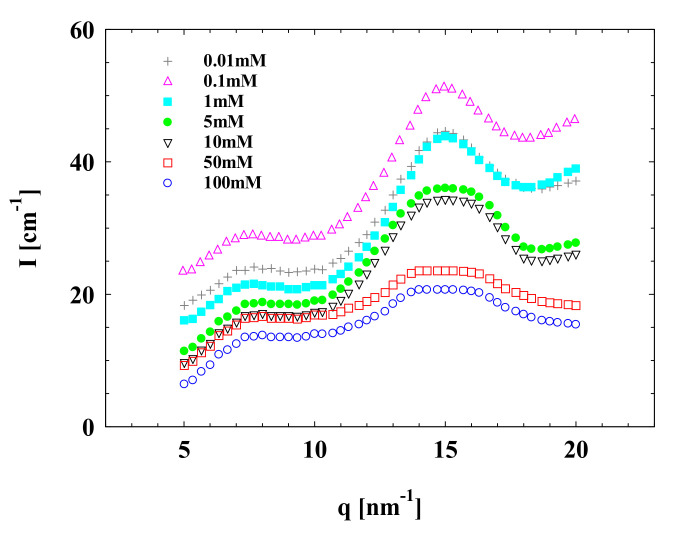
WAXS data of concentrated SPB dispersion at different Al^3+^ concentrations; SPB concentration = 12 wt%, pH = 7.

Changes in the microstructure of the concentrated SPB upon changing Al^3+^ were featured by the local ordered structure peak at around 16 nm^−1^ in the WAXS spectra, as shown in Figure 8. This peak was relatively gentle at both low and relatively high salt concentrations, which is probably due to the fact that at low Al^3+^ concentrations, only part of the carboxyl groups in PAA chains were ionized, leading to very weak interactions among SPBs. However, this began to shield the electrostatic interaction between polyelectrolyte chains, causing shrinkages among the PAA brushes, when the Al^3+^ concentration approached 5 mM. Unlike to Na^+^ and Mg^2+^, when the Al^3+^ concentration increased to 0.1 mM, more carboxyl groups were dissociated, leading to a more obvious local ordered structure peak. At this state, the PAA chains were almost completely charged, and the local ordered structure peak was the largest (Figure 8). This means that the SPB dispersion has the most stable and highest locally ordered structure in this situation. Finally, when the Al^3+^ concentration was adjusted to 100 mM, almost no local ordered structure peak could be observed. Because the arrangement of PAA chains among SPB particles is relatively stable, it is hard for Al^3+^ to enter the net-like local ordered structure among PAA chains, although some space still exists among them. It seems that the local ordered structure is more sensitive to the counterions with higher valence because of their stronger effect on electrostatic interactions. 

### 3.3. Rheology

SAXS and WAXS are able to characterize the interaction and local ordered structures among concentrated SPB dispersions from the microscopic level, while rheology research of SPB dispersion can characterize the interaction and local ordered structures among concentrated SPB dispersions from the macroscopic level. 

**Figure 9:**
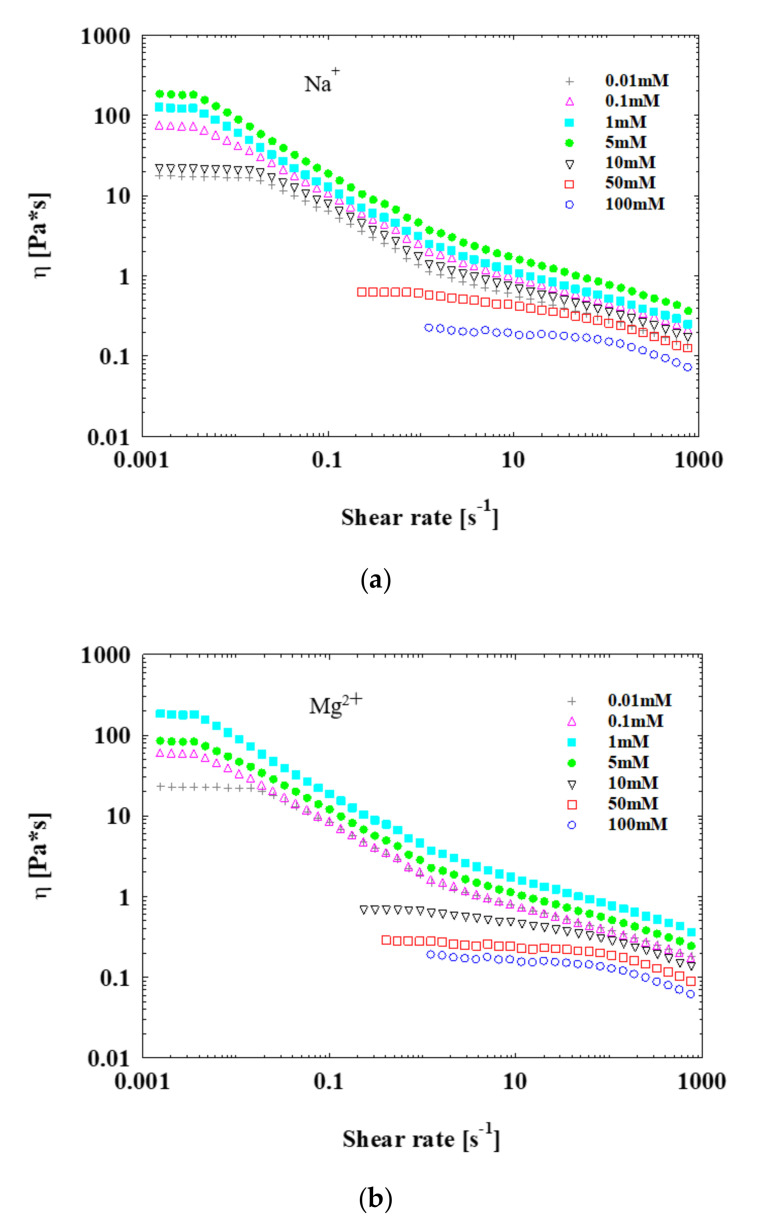

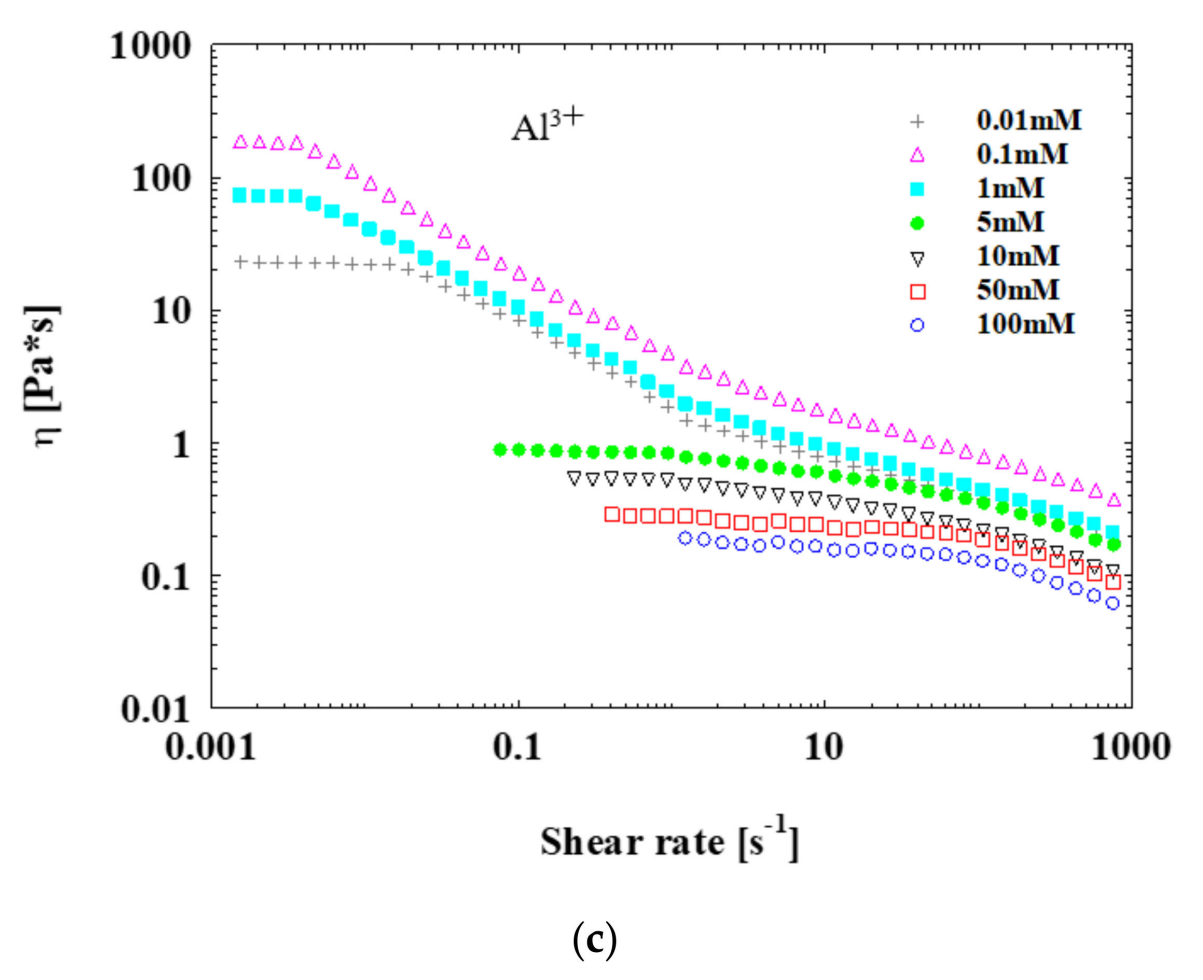
Relationship between viscosity and shear rate of concentrated SPB dispersion at different Na^+^ (**a**), Mg^2+^ (**b**) and Al^3+^ (**c**) ion concentrations; SPB concentration = 12 wt%, pH = 7.

In this context, the steady and dynamic rheological measurements were performed on SPB dispersions in the presence of Na^+^, Mg^2+^ and Al^3+^ concentrations ranging from 0.01 to 100 mM (Figure 9). In all experiments, the SPB concentration was maintained at 12 wt%, and the pH was 7. Upon increasing the Na^+^ concentration from 0.01 to 5 mM, the zero-shear viscosity increased by nearly 10 times (Figure 9). Moreover, with further increase in the Na^+^ concentration to 100 mM, the zero-shear viscosity decreased to 0.1 Pa·s. This confirms the formation of a strong interaction with a net-like structure among SPBs when they are close to a critical distance with a concentration of around 12 wt%, and the counterions as a bridge formed a local ordered structure among PAA chains. This result of the rheology method is consistent with the observations made via SAXS and WAXS.

The rheological properties of SPB dispersion are strongly affected by Mg^2+^ (Figure 9). Slightly different from the case of Na^+^, the zero-shear viscosity increased obviously when the Mg^2+^ concentration elevated from 0.01 to 1 mM, while it leveled off when the Mg^2+^ concentration further increased to 100 mM. The significant enhancement appeared as the Mg^2+^ concentration changes from 0.01 to 1 mM, which is consistent with the trend of the structure factor peak in SAXS spectra and the local ordered structure peak in WAXS spectra. Because the Mg^2+^ concentration affects the degree of dissociation of the carboxyl group, the amount of charge strongly depends on the Mg^2+^ concentration. As a result, the associations among PAA chains from the neighbored SPBs bridged by counterions are enhanced, and a net-like local ordered structure forms, thereby leading to high viscosity. In this way, the counterions influence the interaction among PAA chains. 

Regarding Al^3+^, the maximum of viscosity appears when the concentration is 0.1 mM. At higher Al^3+^ concentrations (>5 mM), the weak electrostatic interactions among PAA chains lead to the low viscosity of SPB dispersion. The PAA chains start to shrink due to the fact that the enhanced counterions decrease the electrostatic interaction among SPBs when the Al^3+^ concentration is adjusted from 0.1 to 100 mM. In the rheological data, a sharp drop in viscosity can be observed, and the SPB dispersion sample changes from a gel-like state to a liquid state. 

**Figure 10:**
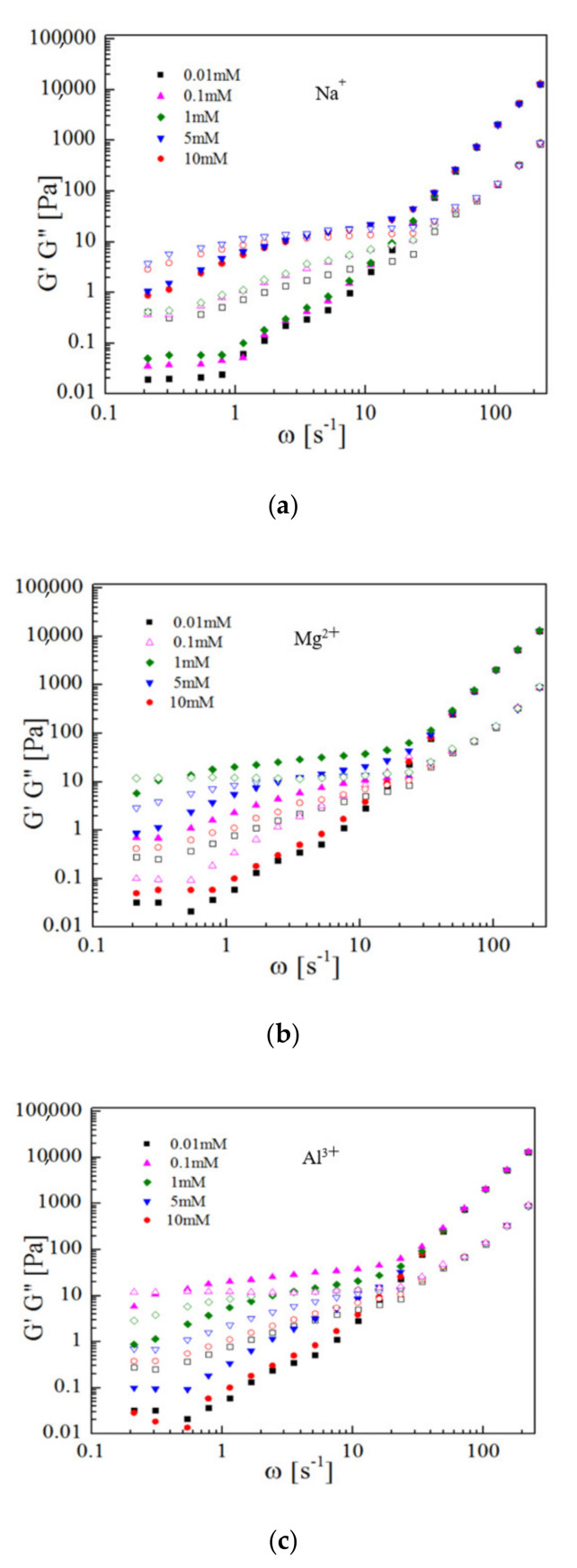
Storage modulus G′ (solid symbol) and loss modulus G″ (open symbol) as functions of frequency at different Na^+^ (**a**), Mg^2+^ (**b**) and Al^3+^ (**c**) concentrations; SPB concentration = 12 wt%, pH = 7.

Figure 10 shows the relationship between storage modulus G′ (solid symbol) and loss modulus G″ (open symbol) as functions of frequency at different Na^+^, Mg^2+^ and Al^3+^ concentrations. The concentration of SPB dispersion was kept constantly at 12 wt% and the pH = 7. As mentioned above, counterions can strongly affect the interaction among concentrated SPB dispersion. These changes are also reflected in dynamic rheological experiments. When pH = 7, the counterions can, to an extent, act as a bridge to promote the dissociation degree of carboxyl groups, leading to a stable net-like structure among PAA chains nearby SPBs. However, salt can also lead to the screening of electrostatic interactions. These two opposite effects of the counterions on the electrostatic interactions result in the appearance of the maximum of *G′* and *G″* upon increasing the concentration of counterions (Figure 10). Thus, the conclusion from the rheology experiment is in good agreement with that of the SAXS and WAXS experiments. The effects of counterion concentration on the appearance of the maximum of *G′* and *G″* are the same as those of the structure factor peak in SAXS spectra and the local ordered structure peak in WAXS spectra.

The phenomena observed by SAXS, WAXS and the rheological methods confirm that the association among SPBs reflected by the structure factor peak in SAXS and local ordered structure peak in WAXS spectra should be driven by the interactions among PAA chains, which can be affected by the counterions.

## 4. Conclusions

The effects of concentration and valence of counterions on the interactions among concentrated SPB dispersion are systematically investigated using SAXS, WAXS and rheological methods. The so-called “structure factor peak” in SAXS curves and the “local ordered structure peak” in WAXS curves reflect the interactions among concentrated SPBs. Both of these two peaks became more significant at moderate strength of counterions, a result which is consistent with our previously reported “polyelectrolyte peak”. The fluctuation of theses peaks can reflect the strength of interaction among concentrated SPBs, which is caused by local ordered structure among PAA chains of neighbor SPBs. Interestingly, the level of local ordered structure can also be compared at the macroscopic level by rheology. The changes in viscosity, the loss modulus and storage modulus derived from differences in strengths of counterions confirm that the local ordered structure reflected by the structure factor peak in SAXS and the local ordered structure peak in WAXS spectra is driven by interactions among concentrated SPB. Through SAXS, WAXS and rheology, we uncovered the formation mechanism of the local ordered structure among PAA chains in the overlapped area of adjacent SPBs, which are bridged by counterions. In contrast, excessive counterions shielded the electrostatic interaction among PAA chains and destroyed the local ordered structure. This work enriches our understanding of the assembly behaviors of concentrated polyelectrolyte particles under the effect of counterions and can serve as the foundations for SPB applications.

## Data Availability

Not applicable.
